# One health laws, policies and governance in Uganda: What's on paper and what's really happening^☆^

**DOI:** 10.1016/j.onehlt.2026.101419

**Published:** 2026-04-18

**Authors:** Laura Ferguson, Musa Sekamatte, Michelle E. Anderson, Collins Agaba, Mercy Haumba, Margarita Kolchina, Dennison Kizito, Janet Seeley, Julius J. Lutwama, on behalf of the COHRIE Uganda Group

**Affiliations:** a Institute on Inequalities in Global Health, University of Southern California, USA; b National One Health Platform, One Health Coordination Office, Ministry of Health, Kampala, Uganda; c Department of Arbovirology, Emerging and Re-emerging Infectious Diseases, Uganda Virus Research Institute, Entebbe, Uganda; d University of Southern California, USA; e London School of Hygiene & Tropical Medicine, London, UK

**Keywords:** One health, Zoonoses, Governance, Implementation, Africa

## Abstract

This paper explores how current national legal, policy and governance structures have affected implementation of Uganda's original National One Health Strategic Plan (NOHSP), with the aim of identifying opportunities for strengthening Uganda's approach to One Health moving forward. We conducted a legal and policy review between August 2022 and May 2023 to assess Uganda's regulatory and institutional landscape for operationalizing One Health. To understand One Health implementation in practice, we conducted 68 semi-structured interviews with participants representing the agriculture, environment, health, policy, and veterinary sectors. The patchwork of antiquated and unrelated laws and policies underpinning Uganda's current NOHSP leaves stakeholders with no legal mandate for implementation. The NOHSP established national-level coordination mechanisms, but across sectors and administrative levels, interview participants indicated a lack of clarity on the existence or structure of subnational One Health mechanisms and their interaction with national structures. Although the NOHSP emphasizes multisectoral collaboration, it lacks specific guidance on how such coordination should be implemented. Interview data indicate that, typically, collaboration is catalyzed by outbreaks, where sectors come together to devise strategies tailored to the immediate crisis rather than based on established protocols. Across all sectors, interview participants pointed out several persisting capacity issues that impact their ability to engage in One Health activities, including understaffing, inadequate funding, and insufficient equipment and facilities. Any One Health accountability measures were primarily described as dictated by donor procedures and sector-specific annual reports, rather than One Health guidelines. While technical guidance for One Health exists at global, regional and sub-regional levels, it often overlooks the reality that One Health is a new paradigm being implemented within pre-existing legal and policy frameworks with a dominant history of sectoral divisions of responsibility, budgets and activities. Operationalizing One Health at a national and subnational level will not only require structural adaptations but also shifts in institutional mindsets.

## 1 Introduction

Over 70% of the pathogens that affect humans are thought to be zoonotic and viral in nature [1], highlighting the urgency of addressing the human, animal, nature interface. While the conceptual linkages between the health of humans, animals and the planet are clear, implementing a One Health approach is challenging as it requires bridging professional and programmatic silos within both government and non-government organizations across diverse sectors, including health, agriculture, environment and wildlife [2], [3]. The COVID-19 pandemic, caused by a virus with zoonotic origins, demonstrated how zoonotic pathogens can rapidly escalate into global health crises and underscored the urgent need for a One Health approach.

Recent global health governance reforms, including amendments to the International Health Regulations in 2024 and the 2025 Pandemic Agreement, have brought One Health more firmly onto the international agenda, recognizing the need to address the human-animal-environment interface to prevent future pandemics and highlighting the need for cross-sector coordination [4], [5].

The World Health Organization (WHO) has recognized that robust governance structures are the foundation of successful One Health, guiding how we integrate scientific evidence, policy and practice [6]. Strong governance includes clear laws and policies, adequate funding, stakeholder and community engagement and effective cross-sectoral coordination [7]. In the context of One Health, laws and policies govern human interactions with animals and nature, shaping prevention and response structures, potential exposures to zoonotic pathogens, and subsequent access to any needed services and interventions to prevent disease outbreaks.

The African Union has prioritized One Health, establishing a ‘One Health Coordination Group’ to lead the development of a continental strategy on One Health and working with Member States to coordinate negotiations within the UN and to support One Health across the continent [3], [8]. Similar challenges to operationalizing One Health have been noted across Africa, including gaps in political commitment, the skilled workforce, domestic financing and the legal frameworks needed to strengthen coordination, collaboration, and communication among relevant stakeholders [9]. At the sub-regional level, the East African Community has developed a Regional One Health Strategy (2022–2027) and facilitated Memoranda of Understanding between Member States to enhance cross-border collaboration on zoonoses. These initiatives present opportunities to bolster One Health approaches through regional cooperation [10].

The Government of Uganda, a country with a long history of zoonotic outbreaks, has recognized the need for a One Health approach. The National One Health Framework (2016) was formalized through a memorandum signed by the collaborating line ministries: Health (MOH); Agriculture, Animal Industries, and Fisheries (MAAIF); Water and the Environment; and the Uganda Wildlife Authority. The Framework established the National One Health Platform (NOHP) to oversee and coordinate these ministries' implementation of One Health. It also laid the groundwork for the first National One Health Strategic Plan (NOHSP; 2018–2022), developed to operationalize One Health. Implementation of the NOHSP is overseen by the National One Health Platform (NOHP), which includes the One Health Technical Working Group^1^ and the Zoonotic Disease Coordination Office,^2^ serving as its secretariat through the One Health Coordination Office. The Government is working on a new NOHSP.

This paper explores how the current legal, policy and governance structures have affected implementation of Uganda's original NOHSP, with the aim of identifying opportunities for strengthening Uganda's approach to One Health moving forward. The data for this paper are drawn from a multidisciplinary research project aimed at improving the understanding and control of zoonotic disease using enhanced surveillance systems and a One Health approach at the human-animal-wildlife interface in Uganda's cattle corridor [11].

## 2 Methods

### 2.1 Legal and policy review

We conducted a legal and policy review between August 2022 and May 2023 to assess Uganda's regulatory and institutional landscape for operationalizing One Health. First, we gathered information on national laws, policies, strategies, and government frameworks relevant to public health, veterinary services, agriculture, and environmental protection. We reviewed Uganda's NOHSP and National Antimicrobial Resistance Action Plan to extract all references to domestic laws and policies in these guiding documents. We searched for publicly available government frameworks, guidelines, and legal documents across relevant sectors and consulted the FAOLEX database^3^ to identify additional relevant national legal instruments. We identified assessments, evaluations, and reports related to One Health policy implementation and institutional capacity in Uganda. Ugandan team members contributed additional documentation, including some which is not publicly available.

Relevant laws, policies, and guidelines were reviewed and analyzed thematically, with key data extracted on legal mandates, institutional responsibilities, intersectoral coordination mechanisms, and accountability structures. This work informed the design of key informant interview (KII) guides, helping to ground interviews in the current legal and policy landscape.

### 2.2 Key informant interviews

To understand how Uganda's legal and policy environment supports or hinders One Health implementation in practice, we conducted 68 semi-structured KIIs. Interview participants were implementers at national and district levels, selected from six study districts across different subregions and national-level institutions involved in One Health activities, reflecting the study's focus on governance and implementation processes. They were purposively selected to ensure representation from the agriculture, environment, health, policy, and veterinary sectors.

District-level KIIs were conducted between October 2022 and March 2023, coinciding with the broader study's household survey phases. National-level KIIs took place between April and May 2023. Interviews were conducted in English by four interviewers, fluent in English and trained in qualitative data collection methods. The interviews were audio recorded and transcribed verbatim in English. Transcripts were reviewed for completeness before being uploaded into NVivo for coding and analysis. The research team developed a codebook based on the study objectives, which was refined iteratively. Transcripts were thematically coded, and emergent themes were discussed. Analytic memos were used throughout the coding and analysis process to support interpretation of findings.

Table 1 summarizes participant characteristics:

**Table 1:** Participant characteristics.

Gender⁎	Totals
Male	56
Female	12
***Sector affiliation***	
Agriculture	4
Environment	15
Health	18
Policy⁎⁎	9
Veterinary	22
***Geographic study area***	
Bwindi Mgahinga	5
Kidepo Valley	3
Lake Mburo-Nakivale	12
Murchison Falls	9
Pian-Upe	9
Queen Elizabeth	8
National level⁎⁎⁎	22

⁎ The gender imbalance of our sample reflects an imbalance in the One Health workforce.

⁎⁎ While many participants were involved in policymaking, the ‘policy’ category refers to those in political or administrative roles, such as District Vice Chairpersons, Chief Administrative Officers, and Resident District Commissioners, whose positions do not fall under Health, Veterinary, Agriculture, or Environment.

⁎⁎⁎ National stakeholders include 19 national and 3 international actors, with the latter group classified as ‘national’ to protect anonymity.

### 2.3 Integrated data analysis

Findings from the document review and interview data were analyzed jointly. This allowed us to triangulate information from legal and policy frameworks with lived experiences and insights from those involved in One Health implementation, providing insight into both the formal structures and the operational realities shaping Uganda's One Health landscape.

## 3 Findings

This section presents our primary findings, organized thematically: One Health structures (national and subnational), political commitment, investments, capacity building (human resources and infrastructure), awareness of One Health, collaboration, and accountability. Where appropriate, selected interview quotes, attributed to participants using study identification numbers, are included to illustrate key points.

### 3.1 One health structures

#### 3.1.1 National

The NOHSP outlines Uganda's approach to operationalizing One Health through existing structures. It refers to a range of laws and policies that underpin it, including the Public Health Act (1962), the Local Government Act (1997), the Animal Diseases Act (1964, partially revised in 2006), the Disaster Preparedness and Management Policy (2010) and the Health Sector Investment Plan (2015–2020). The NOHSP also references international law, including the 2005 International Health Regulations and World Organization for Animal Health Veterinary Legislation. Many other laws, policies, and strategies across sectors are also relevant to One Health, including 19 laws and six policies within livestock and agriculture, six laws and one policy on health security, and five laws and two policies in the wildlife sector.

These laws and policies mostly predate the concept of One Health and do not mandate the type of cross-sector collaboration it requires; no law or policy explicitly mandates the activities outlined in the NOHSP. Thus, the NOHSP relies on a patchwork of international and domestic guidelines that reference related issues but lack coordination. Because the strategy lacks the authority to establish new mandates or structures, it calls for integrating the One Health approach into existing sectoral work, but without allocating additional budget.

The NOHSP provides a national framework and goals for collaboration and implementation across sectors and administrative levels. However, interview data reveal a perceived imbalance in influence among government agencies driving the One Health agenda, with the MOH and MAAIF reportedly having greater influence. Most participants believed national-level One Health structures existed, though few provided examples.

#### 3.1.2 Subnational

The NOHSP does not address subnational One Health mechanisms or detail their governance and implementation responsibilities. Some listed activities hint at this but lack detail. For example, one objective aims to institutionalize One Health within the national government, which includes establishing an emergency fund for outbreak response and mechanisms through which stakeholders can access the fund, but there is no direction for operationalizing this.

Across sectors and administrative levels, interview participants indicated a lack of clarity on the existence or structure of subnational One Health mechanisms and their interaction with national structures. The most common perception is that coordination remains centralized, which is detrimental to effective subnational-level actions.

### 3.2 Political commitment

The NOHSP emphasizes the need for high-level commitment across government levels and aims to ensure stakeholder participation and resource mobilization to support NOHP bodies and mainstream One Health across sectors.

However, two national-level policy participants independently stated there is no political will to implement the activities outlined in the NOHSP, citing different leadership priorities, lack of understanding of how One Health supports existing goals, and the tendency to focus on response over prevention.

These views were echoed by other participants: several from the health and veterinary sectors at the national level cited Uganda's decentralized governance structure as a key challenge in ensuring political will. A national-level participant from the MOH stated:

“*We are deployed under public service in a decentralized system, and we don't expect the central government to have oversight or managerial oversight over the different personnel. So, they report to somebody else, and so we only depend on the goodwill for one to participate” (KII30).*

District-level participants framed the issue not as lack of political will but as a result of insufficient facilitation from the national level and a lack of capacity. They suggested that, despite potential challenges due to varying subnational priorities and the need for widespread sensitization, the decentralized structure of Uganda would greatly benefit from a champion for One Health in every district.

### 3.3 Investments

The NOHSP recognizes the importance of sustainable funding. It includes plans for sourcing funding domestically and from development partners and the private sector. However, interview findings reveal a stark contrast between this vision and the current reality. Participants noted that due to limited government resources, most funds supporting One Health come from international donors. These funds are unevenly distributed, and sectors are reluctant to share resources for collaboration. Participants across sectors and administrative levels stated that most funding relevant to One Health goes toward emergency response and focuses on human health without cross-sector collaboration. Further, while the NOHSP proposes sectoral reallocation of existing budgets to accommodate One Health, interviews made clear this has not occurred; this was most likely due to resource constraints and the absence of legal mandates for such reallocations.

### 3.4 Capacity building

The NOHSP emphasizes the importance of capacity, proposing training, investment in infrastructure, and resource allocation across sectors. However, participants across all sectors pointed out several persisting capacity issues that impact their ability to engage in One Health activities, including understaffing, inadequate funding, and insufficient equipment and facilities.

#### 3.4.1 Human resources

Interview participants at both district and national levels identified a lack of human resources as a critical barrier to implementing One Health, stating that even where laws or policies are in place, many sectors cannot conduct mandated work due to understaffing. Participants from the veterinary sector were among those citing concerns about insufficient personnel and turnover:

*“We also have issues of capacity. Um, you know, for example, we have three technicians who work in our laboratory. We've spent some, I think five or six years training them and getting them to a good level and then out of the blue, one of them resigns, and then you have only two, and yet you need actually 10” (KII38).*

Even in situations where there is an adequate workforce, this must also be supported through funding. A national-level participant described recruiting and training more skilled workers for the agricultural sector but then found that there was no operational budget *(KII44)*. Similarly, a national-level participant from the veterinary sector stated: *“Now there are two things, the ability and capacity; one requires the knowledge, and one requires the money facilitation. For example, if a job is going to be done and you give me my per diem but don't give me fuel, [...] I will not be capable of doing that job” (KII45).*

#### 3.4.2 Infrastructure

Another capacity-related barrier to implementation is the lack of adequate materials or facilities for One Health activities, with participants noting shortages of vaccines, reagents, equipment and transportation*.* A veterinary participant who works at the national level described inadequate abattoir facilities: “*We lack sanitizers*” and “*in case there is an outbreak of a dangerous disease, e.g., anthrax, we lack transporting the carcass from an area without contaminating adjacent areas, we don't have means of transporting*” *(KII47).*

An agricultural participant working at the district level discussed inadequate laboratory capacity: “*We do not have adequate facilities to diagnose these epidemics [that are] mostly hemorrhagic*” *(KII26).* Diagnostic infrastructure remains centralized, with tests for zoonoses carried out in Entebbe, which is more than a day's drive from some places. This results in high costs and long delays in delivering test results, particularly in remote regions.

### 3.5 Awareness of one health

The NOHSP's objectives include creating knowledge, awareness, and support for One Health by developing and implementing a public communication plan; engaging in advocacy, communication, and training; and sensitizing interest groups on their roles and responsibilities.

National participants across sectors generally demonstrated a comprehensive understanding of One Health concepts and goals, while many district-level participants described a superficial or incomplete understanding of One Health. District-level participants within agriculture had a notably stronger understanding of One Health and its importance than their counterparts in other sectors.

While the strategy only briefly mentions the role of educational institutions, several participants highlighted institutions like the Uganda Virus Research Institute and Makerere University as influential in spreading awareness and collaborating with ministries on One Health activities.

### 3.6 Collaboration

Although the NOHSP emphasizes multisectoral collaboration, it lacks specific guidance on how to implement such coordination. Interview data indicate that, typically, collaboration is catalyzed by outbreaks, where sectors come together to devise strategies tailored to the immediate crisis rather than based on established protocols. Participants across sectors noted that emergency collaborations primarily involve only the health and veterinary sectors.

The lack of a One Health policy framework and of specific directives in the NOHSP were cited as major barriers to sustained collaboration. Participants stressed the need for clear directives to guide joint activities. A district veterinary sector participant commented: *“The collaboration is on paper, it's in theory, and that is why the monitoring is also not effective.” (KII02).* Similarly, a national agricultural sector participant suggested: *“Of course, one way of promoting collaboration is establishing some more legal framework [...] it's a way of also of improving the collaboration because each of the sectors will know why they need to collaborate.” (KII34).*

Some participants cited stakeholders' lack of inclination to collaborate, possibly reflecting the need for further sensitization efforts on how One Health supports existing sector goals.

While these constraints hinder sustained collaboration, participants also identified sources of support that help facilitate coordination and other One Health principles in practice. The NOHP is a strong group at the national level who continue to advocate for investment in and commitment to One Health. Several participants also noted the importance of international partners, such as UN agencies including WHO, in advising and strengthening collaborations and funding mechanisms. It can be inferred that these partners play a more supportive role in practice than outlined in the strategy.

### 3.7 Accountability

Across sectors, the interview findings underscored the lack of monitoring and evaluation of One Health activities, except, as with many other things, in the context of emergencies or disease outbreaks. Participants emphasized that the activation of One Health structures during outbreaks reflects the fact that coordination is largely crisis-driven rather than routine. Several respondents described this pattern as a systemic feature of Uganda's multisectoral architecture, noting that the mechanisms exist but lack the mandates, incentives, and resources needed to function continuously outside of emergency contexts.

More examples of One Health-related monitoring and evaluation were cited at the national level than the district level. A national-level participant from the policy sector pointed out a lack of funding, budget and coordination available for district monitoring and evaluation (KII50), while a health sector participant noted that districts have considerable discretion in how they interpret or prioritize One Health responsibilities, resulting in wide variation in reporting practices and follow-through. This decentralized decision-making, while critical for ensuring locally appropriate action, was described as contributing to inconsistent levels of monitoring and coordination across districts.

The health and veterinary sectors highlighted existing monitoring and evaluation efforts for reporting back to external donors; accountability measures were primarily dictated by donor procedures and sector-specific annual reports, rather than One Health guidelines. Within this, participants cited activities such as joint sector reviews, audits, and quarterly meetings to review accomplishments within their budget frameworks. Notably, only participants from the policy sector mentioned the Joint External Evaluation on health security outlined in the NOHSP.

The findings from the interviews suggest that an approach to monitoring and evaluation has not yet been fully developed, but the strategic plan indicated that this would be included in future efforts.

## 4 Discussion

With collaboration across many different sectors and at all levels from global to local, the coordination required for effective One Health is daunting. Laws and policies can establish the parameters for this work, including mandating the governance structures within which it should occur, but any legal and policy framework is only as good as its implementation. In this paper, we sought to understand not only the content of laws and policies relevant to One Health in Uganda but also, through interviews, the degree to which and reasons why they are (and are not) being implemented, with the aim of informing policymakers, implementers and development partners seeking to strengthen One Health coordination and governance.

The patchwork of antiquated and unrelated laws and policies underpinning Uganda's current NOHSP leaves stakeholders with no legal mandate for implementation. Although currently stalled due to a lack of funding, the Ugandan government is currently working on a One Health policy, which, it is hoped, can strengthen the mandate for implementing the next NOHSP. While the Offices of the President and the Prime Minister have championed the policy, some officials in key ministries have expressed hesitation, questioning the need for a standalone One Health policy rather than integrating components into existing sectoral policies. In Uganda, ongoing internal challenges such as inter-ministerial competition over hosting the One Health Coordination Office and the lack of a corresponding budget, regardless of its placement, reflect deeper constraints in political commitment.

There is a stark difference between the NOHSP's intended funding strategy and the practical challenges and perceptions reported by participants. Alimi and Wabacha [3] recently wrote about the importance of strong political commitment, financial investments and institutionalized responses for creating the policy and practice changes needed for effective One Health. One Health requires unified, transparent and large-scale funding, while funders often prefer funding disease-specific programs within a single discipline. In the current context of funding cuts and competing priorities, it remains unclear how One Health will be prioritized. Dedicated resources for One Health activities that are formally allocated through legislation or policy could help effectively support One Health implementation. Some examples exist of creating a unifying funding structure for One Health [13], [14], [15], [16] and of using local funding and infrastructure as the basis for One Health actions [12], [13], [15].

Participants pointed to relational and institutional dynamics that constitute challenges to collaboration such as conflicts of interest, coordination gaps, and resistance to the One Health approach based on interpersonal tensions, power struggles, and biases around leadership roles. These dynamics contribute to mistrust and undermine willingness to collaborate, even when structures exist to support joint action. The shortcomings in intersectoral collaboration, particularly at the ground level, documented in this study resonate with the global acknowledgement that the effective implementation of One Health is hindered by *“professional segregation with limited cross-sectoral working, inadequate representation of some sectors, disjointed legislative schemes, a lack of data sharing and transparency, an absence of multisectoral coordination mechanisms, siloed budgets and decision-making processes, and a lack of robust regulatory frameworks, legal support, mandates and enabling policies”* [7].

Governance tends to function more effectively when all actors have relatively equal power, which has not traditionally been the case across government sectors in Uganda. Yet, positive examples of intersectoral collaboration in other country contexts demonstrate that joint action is possible and provide models from which lessons might be drawn [12], [13], [14]. Across Africa, the need to strengthen political commitment, legal frameworks and domestic financing has also been noted [9].

Study participants highlighted a tendency to prioritize response over prevention, despite the latter being central to One Health. Participants linked this issue to a lack of capacity, funding, a One Health legal instrument, and sensitization around how One Health supports sectoral goals. Constraints such as limited expertise, resources, infrastructure, and political will– concerns voiced across all sectors− prevent coordinated action on complex challenges. With subnational participants suggesting that implementation and coordination are limited due to insufficient facilitation from national stakeholders, and national participants instead stating that lack of political will is the constraint, everyone seems to be looking upstream for guidance, authority and support for this work.

The low levels of understanding of One Health concepts reported at subnational level may also hinder intersectoral collaboration; the relatively high knowledge of subnational workers in the agricultural sector might provide a learning opportunity for how to further improve knowledge across other sectors. In addition to sectoral representation, imbalances in workforce composition, such as the gender distribution reflected in our sample, illustrate another dimension of uneven participation that may influence how One Health responsibilities are negotiated in practice.

Despite these challenges, Uganda has a strong foundation on which to build the One Health response. The National One Health Platform provides a structure for governance, which could be extended down to the district level. Evidence that sectors have collaborated effectively during outbreaks suggests that such collaboration could also function on a more sustained basis. And the strength of local institutions such as Makerere University and the Uganda Virus Research Institute could be further leveraged to maximize the use of established technical expertise for One Health and develop the next generation of talent. Furthermore, despite shortcomings in implementation, the content of the NOSHP is strong and can serve as a starting point for the next iteration of the strategic plan as well as the content of the planned One Health policy. Lastly, evident commitment to One Health from the African Union and the East African Community provides a supportive framework within which Uganda can continue to strengthen political commitment and collaboration.

## 5 Conclusion

Several actionable recommendations emerge from this study including the need for clearer legal mandates for multi-sectoral coordination and to develop a stronger, unified national system for routine surveillance, monitoring and evaluation for One Health. At the subnational national level formalizing the district One Health committees and embedding One Health in district development plans would strengthen local ownership and accountability. Sustainable financing approaches are also required, including creation of dedicated domestic budget lines for One Health within sectoral ministries and pooled contingency funds to support rapid joint response to zoonotic threats and reduce reliance on donor-driven funding sources. Continued sensitization at all levels from the national to the local will also be needed to garner cross-sectoral political commitment to prioritizing One Health as well as demand for this from communities.

This paper draws on data from Uganda, but broader literature suggests that its findings might also be useful to inform One Health approaches elsewhere. While technical guidance for One Health exists at global, regional and sub-regional levels, it often overlooks the reality that One Health is a new paradigm being implemented within pre-existing legal and policy frameworks with a dominant history of sectoral divisions of responsibility, budgets and activities. Operationalizing One Health at a national and subnational level will not only require structural adaptations but will also require shifts in institutional and individual mindsets.

## CRediT authorship contribution statement

**Laura Ferguson:** Writing – review & editing, Writing – original draft, Validation, Supervision, Project administration, Methodology, Investigation, Funding acquisition, Formal analysis, Conceptualization. **Musa Sekamatte:** Writing – review & editing, Validation. **Michelle E. Anderson:** Writing – original draft, Formal analysis. **Collins Agaba:** Writing – review & editing, Validation, Supervision, Investigation, Conceptualization. **Mercy Haumba:** Writing – review & editing, Investigation. **Margarita Kolchina:** Writing – review & editing, Formal analysis. **Dennison Kizito:** Writing – review & editing, Project administration. **Janet Seeley:** Writing – review & editing, Validation, Supervision, Methodology. **Julius J. Lutwama:** Writing – review & editing, Funding acquisition.

## Declaration of competing interest

The authors declare that they have no known competing financial interests or personal relationships that could have appeared to influence the work reported in this paper.

## Data Availability

Anonymized data will be made available on reasonable request to the corresponding author.

## Footnotes

1 The OHTWG, comprising 36 members from various government and international stakeholders, provides oversight, coordination, and technical guidance, with its chair rotating between ministries every six months.

2 The ZDCO, serving as the NOHP secretariat, includes two members from each coordinating ministry (MOH, MAAIF, MWE, UWA) and manages administrative and secretarial duties for the plan's implementation.

3 The FAOLEX database is an online repository of national legislation, policies, and international agreements on food, agriculture, and natural resources, maintained by the Food and Agriculture Organization (https://www.fao.org/faolex/en/).
